# Genetic Risk in Families with Age-Related Macular Degeneration

**DOI:** 10.1016/j.xops.2021.100087

**Published:** 2021-12-06

**Authors:** Anita de Breuk, Yara T.E. Lechanteur, Thomas J. Heesterbeek, Sascha Fauser, Caroline C.W. Klaver, Carel B. Hoyng, Anneke I. den Hollander

**Affiliations:** 1 Department of Ophthalmology, Donders Institute for Brain, Cognition and Behaviour, Radboud University Medical Center, Nijmegen, The Netherlands; 2 Department of Ophthalmology, University Hospital of Cologne, Cologne, Germany; 3 F. Hoffmann-La Roche, Basel, Switzerland; 4 Department of Ophthalmology, Erasmus Medical Center, Rotterdam, The Netherlands; 5 Department of Epidemiology, Erasmus Medical Center, Rotterdam, The Netherlands; 6 Institute of Molecular and Clinical Ophthalmology, Basel, Switzerland

**Keywords:** Age-related macular degeneration, Complement factor H, Complement factor I, Complement system, Genetic risk score, AMD, age-related macular degeneration, CCP, complement control protein, CFH, complement factor H, CFI, complement factor I, CI, confidence interval, CIRCL, Cologne Image Reading Center and Laboratory, CNV, choroidal neovascularization, GA, geographic atrophy, GRS, genetic risk score, GWAS, genome-wide association study, IQR, interquartile range, RC, Rotterdam Classification, SE, standard error

## Abstract

**Purpose:**

To determine the contribution of common and rare genetic risk variants in families with age-related macular degeneration (AMD).

**Design:**

Case-control study.

**Participants:**

A family cohort (355 affected and 342 unaffected family members from 144 families with AMD) and an unrelated case-control cohort (1078 patients, 952 controls), recruited from the European Genetic Database.

**Methods:**

Genetic data of both cohorts were filtered for carriership of rare genetic variants in the coding and splice-site regions of the complement factor H (*CFH*) and complement factor I (*CFI*) genes, and 52 AMD-associated variants were extracted for calculation of genetic risk scores (GRS). To compare GRSs between familial and nonfamilial rare *CFH* and *CFI* variant carriers and noncarriers and between AMD disease stages, we performed a 2-way analysis of variance, with Bonferroni correction for multiple testing. Within families with AMD carrying rare *CFH* and *CFI* variants, we analyzed segregation patterns by calculating the proportion of affected among carriers.

**Main Outcome Measures:**

GRSs and segregation of rare *CFH* and *CFI* variants.

**Results:**

We observed higher GRSs in familial versus nonfamilial individuals without rare *CFH* and *CFI* variants: mean GRS, 1.76 (standard error [SE], 0.08) versus 0.83 (SE, 0.03; *P* < 0.001). In 51 of 144 families (35.4%), rare *CFH* and *CFI* variants were identified. Within the AMD family cohort, carriers of rare *CFH* and *CFI* variants showed lower GRSs compared with noncarriers (mean GRS, 1.05 [SE, 0.23] vs. 1.76 [SE, 0.08]; *P* = 0.02). The proportion of affected family members with a high GRS was 57.3% (176/307). Of the affected family members with a low or intermediate GRS, 40.0% carried rare *CFH* or *CFI* variants. Among carriers of 11 rare *CFH* or *CFI* variants, the proportion affected by AMD was more than 75%.

**Conclusions:**

Genetic risk in families with AMD often is attributed to high GRSs based on common variants. However, in part of the families with a low or intermediate GRS, rare *CFH* and *CFI* variants contributed to disease development. We recommend computing GRSs and sequencing the *CFH* and *CFI* genes in families with AMD, in particular in the light of ongoing gene-specific clinical trials.

Age-related macular degeneration (AMD) is the leading cause of severe and irreversible vision loss among the elderly in developed countries.^1^^,^^2^ Its pathogenesis involves both genetic and nongenetic risk factors. Evidence for a strong genetic contribution originated from twin studies and family-based studies.^3^, ^4^, ^5^, ^6^ To date, common genetic variants contributing to AMD risk are well defined through genome-wide association studies (GWASs).^7^ Because of the low allele frequency of rare genetic variants, identifying significant associations between them and AMD is more challenging. However, with the introduction of next generation sequencing, an increasing number of rare variants have been identified in patients with AMD, mainly in genes involved in the complement system.^8^

Genetic risk for AMD can be evaluated by studying the contribution of common genetic risk variants to the development of AMD. For example, the cumulative effect of the 52 AMD-associated variants as described by Fritsche et al^7^ can be determined by calculating a genetic risk score (GRS).^9^^,^^10^ Alternatively, a smaller subset of well-validated common genetic variants can be used to observe differences in expected and observed numbers of risk alleles to elucidate the contribution of genetic variants common to AMD development. Previous family-based studies demonstrated that in a substantial number of families with AMD, genetic risk can be explained by common genetic variants, but in a subset of families, this is not the case.^11^, ^12^, ^13^, ^14^, ^15^ It has been hypothesized that in the latter families, rare genetic variants contribute to disease development. Indeed, in previous studies, highly penetrant rare variants in the complement factor H (*CFH*) and complement factor I (*CFI*) genes have been observed in families with AMD, and a clustering of specific rare complement variants in families with AMD has been described.^11^^,^^13^^,^^14^^,^^16^, ^17^, ^18^, ^19^, ^20^ However, rare variants do not always fully segregate with the disease in these families, meaning that these variants are not the sole factors that drive disease development. For genetic counseling purposes and for the development of personalized medicine approaches, it is important to determine the contribution of both common and rare genetic variants to AMD, especially considering that clinical trials for AMD targeting the complement pathway are selecting patients based on genotype (https://www.clinicaltrialsregister.eu/ctr-search/trial/2019-003421-22/GB and https://clinicaltrials.gov/ct2/show/study/NCT04246866).

Considering the major influence of genetics on AMD, we hypothesize that either a high GRS based on common variants or the presence of rare highly penetrant variants in complement genes *CFH* and *CFI* contribute to the development of AMD. The purpose of this study was to determine the contribution of common and rare genetic variants in families with AMD. First, we determined the contribution of common variants by comparing GRSs between families with AMD (with and without rare *CFH* and *CFI* variants) and an unrelated case-control cohort. Next, we studied the contribution of rare genetic variants by determining segregation patterns of rare *CFH* and *CFI* variants in individuals from families with AMD.

## Methods

### Study Population

For this study, we selected a family cohort (n = 697 individuals from 144 families with AMD) and an unrelated case-control cohort (n = 2030 individuals) from the European Genetic Database, representing Dutch and German individuals. Patient recruitment took place from October 2004 through October 2019. We split the family cohort into 2 different groups: families with familial AMD, defined as at least 2 first-, second-, or third-degree relatives in the family with AMD (n = 143 patients with early or intermediate AMD, 164 patients with advanced AMD, and 246 family members without AMD from 96 families) and families with only 1 individual affected by AMD within the family (n = 21 patients with early or intermediate AMD, 27 patients with advanced AMD, and 96 family members without AMD from 48 families). Within those groups, we subdivided the families into families carrying rare genetic variants in the *CFH* or *CFI* genes and families without rare genetic variants in the *CFH* or *CFI* genes. The case-control cohort consisted of 451 patients with early or intermediate AMD, 619 patients with advanced AMD, and 952 control individuals of 65 years of age or older. Only individuals without a reported history of AMD in the family, based on self-reported questionnaires, were included in the case-control cohort. The overview of the cohort selection is provided in Figure S1 (available at www.ophthalmologyscience.org). This study was approved by the Medical Ethics Committee of the Radboud University Medical Center, Nijmegen, The Netherlands, and adhered to the tenets of the Declaration of Helsinki. All study participants provided written informed consent.

### Image Acquisition and Grading

Thirty-five–degree color fundus photographs centered on the fovea were obtained using a Topcon TRC 50IX camera or Topcon DRI Triton camera (Topcon Corp., Tokyo, Japan). OCT images were captured using a Spectralis HRA+OCT device (Heidelberg Engineering). In 94.5% of the individuals (2578/2727), grading of the images was performed according to the standard protocol of the Cologne Image Reading Center and Laboratory (CIRCL).^21^ In 4.5% of the individuals (149/2727), images were graded according to the Wisconsin Age-Related Maculopathy Grading System and reclassified into the Rotterdam Classification (RC),^22^, ^23^, ^24^ as described previously. This was the case if CIRCL grading (of the most recent image date) was not available. Individuals were categorized into 3 phenotype groups: no AMD (no signs of AMD or ≤10 small drusen ≤63 μm together with pigmentary changes [CIRCL]; RC grade 0), early or intermediate AMD (≥10 small drusen ≤63 μm together with pigmentary changes, ≥1 intermediate drusen 63–124 μm, or large drusen ≥25 μm [CIRCL]; RC grade 1–3), advanced AMD (choroidal neovascularization [CNV] or geographic atrophy (GA) in at least 1 eye [CIRCL]; RC grade 4).

### Genotyping

Genotyping data were available based on 1 or more of the following genotyping platforms: whole exome sequencing,^25^ customized HumanCoreExome array,^7^ single-molecule molecular inversion probes,^10^ or competitive, allele-specific polymerase chain reaction assays (KASP SNPs Genotyping; LGC Group),^26^, ^27^, ^28^ and was performed as described previously. A GRS was calculated for each individual based on the 52 AMD-associated variants,^7^ which were extracted from the customized HumanCoreExome array^7^ and single-molecule molecular inversion probe datasets.^10^ We used the following formula for GRS calculation: $GRS=\;\overset{52}{\underset{i\;=\;1}{\sum }}\left(G_{i\;}\beta_{i}\right)$, where *G*_*i*_ represents the genotype of variant *i* and β_*i*_ represents the effect size of variant *i* (natural logarithm of the fully conditioned odds ratio of the minor allele of variant *i*), based on the GWAS of the International Age-Related Macular Degeneration Genomics Consortium.^7^ Genotypes were coded as 0, 1, or 2 based on the number of minor alleles. Genotypes of the major risk or protective variants *CFH* rs570618, *CFH* rs10922109, *C2*/*CFB*/*SKIV2L* rs429608, *ARMS2* rs3750846, or *C3* rs2230199 were mandatory to calculate a GRS. If one of those variants was not available, we considered the GRS of that individual as missing. The GRS of the individuals in the case-control cohort were categorized into 3 equal GRS groups, with the follow categories: low (GRS, ≤0.220), intermediate (GRS, 0.221–1.407), and high (GRS, ≥1.408).

Genotyping data were filtered for rare splice-site and protein-altering variants in the *CFH* and *CFI* genes (minor allele frequency, <0.01, based on the non-Finnish European population [Genome Aggregation Database]). Individuals carrying 1 or more rare *CFH* or *CFI* variants were considered carriers, and individuals without any rare *CFH* or *CFI* variants were considered noncarriers. In families with a low GRS, we additionally filtered for the genetic data of rare variants in complement genes *C3* and *C9* and the genes *TIMP3* and *SLC16A8*, because rare variants in these genes previously were described in families with AMD^18^ or because a burden of rare variants in these genes was reported previously in patients with AMD.^7^

### Statistical Analysis

Data were analyzed from April 2020 through April 2021. We analyzed general characteristics of the study cohorts by using Kruskal-Wallis H tests and chi-square tests. Results are presented as medians with corresponding interquartile ranges (IQRs) or numbers with corresponding percentages. *P* values of < 0.05 were considered statistically significant. For pairwise comparisons, we performed chi-square tests and Mann–Whitney *U* tests. We applied a Bonferroni correction for multiple testing. The significance threshold was set at *P* < 0.05 / 3 = 0.0167.

We aimed to evaluate differences in GRSs between familial and nonfamilial AMD and between rare *CFH* or *CFI* variant carriers and noncarriers, and therefore included the 96 families from the family cohort (the familial AMD cohort) and the case-control cohort (the nonfamilial AMD cohort; Fig S1). The 48 families with only 1 affected individual were not included for GRS analysis. Family members younger than 65 years old, without AMD, were excluded for GRS analysis because AMD still could develop in them. Also, individuals without available GRSs were excluded for analysis. Individuals were categorized into 4 groups: (1) familial rare *CFH* or *CFI* variant carriers, (2) familial noncarriers, (3) unrelated rare *CFH* or *CFI* variant carriers, and (4) unrelated noncarriers. We performed a 2-way analysis of variance to compare GRSs among the 4 groups and among the AMD disease stages. Pairwise comparisons were conducted with Bonferroni adjustment for multiple comparisons. *P* values of less than 0.05 were considered statistically significant.

For the analysis of rare *CFH* and *CFI* variants in families with AMD, we included 51 families carrying rare variants in the *CFH* and *CFI* genes (Fig S1) and analyzed segregation patterns of the rare *CFH* and *CFI* variants that were identified in the 51 families. For each of the rare *CFH* and *CFI* variants, we determined the fraction of individuals carrying a given *CFH* or *CFH* variant that manifests AMD:

$$\frac{\text{Number of carriers of variant}\;i\;\text{affected by any AMD}}{\text{Total number of carriers of variant}\;i}$$

Because the early and intermediate AMD stages generally lead to minimal loss of visual acuity, whereas the advanced stages usually result in severe vision loss, we additionally calculated the ratio of carriers affected by advanced AMD to the total number of carriers. Family members younger than 65 years without AMD still can demonstrate AMD characteristics because those individuals had not reached the age at onset of AMD. Considering a substantial proportion of the family cohort is younger than 65 years, we performed a subanalysis by excluding family members younger than 65 years without AMD. This was the case for 13 rare variants. In case we identified only 1 carrier of a specific rare *CFH* or *CFI* variant, segregation analysis could not be determined. All analyses were performed using SPSS software version 22 (IBM Corp., Armonk, NY).

## Results

A total of 697 individuals from 144 families with AMD and 2030 unrelated case and control participants were included in the study. General characteristics of the study cohorts are shown in Table 1.

**Table 1:** General Characteristics of the Study Cohorts

Characteristic	Case-Control Cohort (n = 2030)			Families with ≥2 Affected Individuals (n = 553 Individuals from 96 Families)			Families with 1 Affected Individual (n = 144 Individuals from 48 Families)			*P* Value
	*CFH* or *CFI* Rare Variant Carrier (n = 116)	*CFH* or *CFI* Noncarrier (n = 1914)	*P* Value∗	*CFH* or *CFI* Rare Variant Carrier (n = 101)	*CFH* or *CFI* Noncarrier (n = 452)	*P* Value∗	*CFH* or *CFI* Rare Variant Carrier (n = 30)	*CFH* or *CFI* Noncarrier (n = 114)	*P* Value∗	
Age (yrs)	73 (68–79)	72 (68–78)	0.41	67 (56–77)	65 (57–74)	0.23	58 (48–69)	61 (49–73)	0.25	**<0.001**
Gender			0.22			0.85			0.96	0.89
Male	43 (37.1)	820 (42.8)		43 (42.6)	197 (43.6)		13 (43.3)	50 (43.9)		
Female	73 (62.9)	1094 (57.2)		58 (57.4)	255 (56.4)		17 (56.7)	64 (56.1)		
Disease stage			**0.002**			**<0.001**			0.05	**<0.001**
No AMD	39 (33.6)	913 (47.7)		24 (23.8)	222 (49.1)		16 (53.3)	80 (70.2)		
Early/intermediate	28 (24.1)	417 (21.8)		25 (24.8)	118 (26.1)		4 (13.3)	17 (14.9)		
Advanced	49 (42.2)	584 (30.5)		52 (51.5)	112 (24.8)		10 (33.3)	17 (14.9)		

AMD = age-related macular degeneration.

Gender, age, and disease stage among the 3 study groups were compared using a chi-square test (gender) or a Kruskal-Wallis H test (age and disease stage). *P* values of < 0.05 are considered statistically significant (last column). Post hoc analyses were performed to evaluate differences in gender, age, and phenotype between *CFH* or *CFI* rare variant carriers and *CFH* or *CFI* noncarriers within the 3 study groups by using chi-square tests (gender) or Mann–Whitney U tests (age and disease stage). Because we performed 3 post hoc tests, the significance level was adjusted to 0.0167 (0.05/3; Bonferroni correction for multiple testing). Categorical variables are presented as numbers with corresponding percentages and continuous variables as medians with corresponding interquartile ranges. Significant results appear in boldface.

∗ Adjusted *P* value.

### Genetic Risk Score in Familial Age-Related Macular Degeneration and Unrelated Individuals (Nonfamilial Age-Related Macular Degeneration)

**Figure 1:**
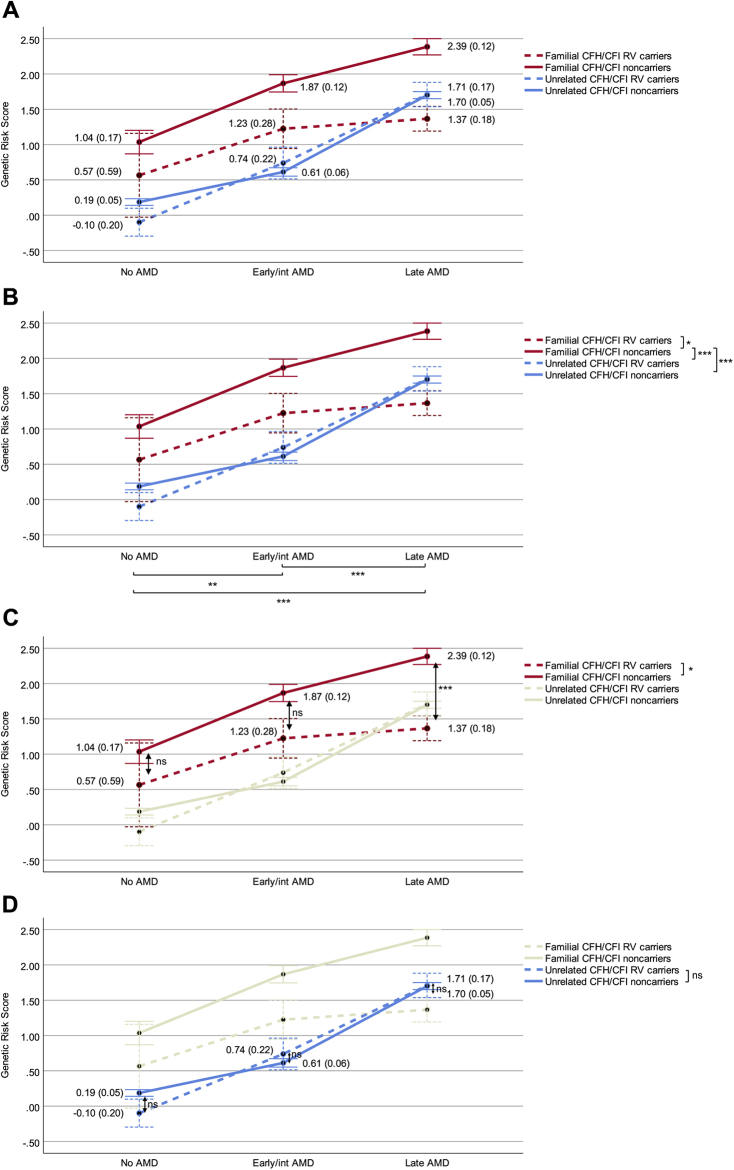
Graphs showing the genetic risk score in familial age-related macular degeneration (AMD) and unrelated individuals. The y-axis represents the estimated marginal mean genetic risk score. The x-axis represents the AMD disease stages. The family cohort is indicated in red, and the unrelated case-control cohort is indicated in blue. Dotted lines represent rare *CFH* and *CFI* variant carriers, and continuous lines represent *CFH* and *CFI* noncarriers. Error bars are ±1 standard error. Numbers within the figures indicate the estimated marginal mean genetic risk scores with corresponding standard errors. A 2-way analysis of variance was performed to compare genetic risk scores among the 4 groups and among the AMD disease stages. A Bonferroni correction for multiple testing was applied for all pairwise comparisons (**B**–**D**). **A**, Genetic risk score for all categories and AMD disease stages. **B**, Pairwise comparisons of AMD disease stages and group categories. **C**, Pairwise comparisons within the family cohort. **D**, Pairwise comparisons within the case-control cohort. ∗*P* < 0.05; ∗∗*P* < 0.01; ∗∗∗*P* < 0.001. int = intermediate; ns = not significant; RV = rare variant.

We performed a 2-way analysis of variance to compare the GRSs between rare *CFH* and *CFI* variant carriers and noncarriers in familial AMD (families with at least 2 affected individuals) and unrelated individuals and between AMD disease stages. Mean GRSs are depicted in Table S1 (available at www.ophthalmologyscience.org) and Figure 1A.

#### Analysis of Variance Main Effects of Age-Related Macular Degeneration Disease Stages and Group Category on Genetic Risk Score

First, we analyzed the main effects of the AMD disease stages and group category on GRS. We observed a significant difference in GRS among individuals without AMD (mean, 0.44; standard error [SE], 0.16), early or intermediate AMD (mean, 1.11; SE, 0.10), and advanced AMD (mean, 1.79; SE, 0.07; *F*(2, 1999) = 38.46; *P* < 0.001). We also observed significant differences in GRSs between familial *CFH* and *CFI* rare variant carriers (mean, 1.05; SE, 0.23), familial noncarriers (mean, 1.76; SE, 0.08), unrelated *CFH* and *CFI* rare variant carriers (mean, 0.87; SE, 0.12), and unrelated noncarriers (mean, 0.83; SE, 0.03; *F*(3, 1999) = 41.35; *P* < 0.001). Furthermore, a significant interaction was found between the group category and AMD disease stages on GRS (*F*(6, 1999) = 3.07; *P* = 0.005), indicating that the effect of group category on GRS depends on the AMD disease stages (Table S2, available at www.ophthalmologyscience.org).

#### Analysis of Variance Pairwise Comparisons of Genetic Risk Score between Age-Related Macular Degeneration Phenotypes and between Group Categories

To gain insight into which specific groups account for the differences in GRS, we performed pairwise comparisons (with Bonferroni correction for multiple testing). When focusing on the AMD disease stages (without taking into account the group category), we observed that individuals with advanced AMD have significantly higher GRSs compared with individuals with early or intermediate AMD (mean difference, 0.68; 95% confidence interval [CI], 0.40–0.96; *P* < 0.001) and individuals without AMD (mean difference, 1.37; 95% CI, 0.95–1.79; *P* < 0.001; Fig 1B; Table S3, section A, available at www.ophthalmologyscience.org). Pairwise comparisons between group categories (without taking into account the AMD disease stages) revealed significantly higher GRSs in familial noncarriers compared with the familial *CFH* or *CFI* rare variant carriers (mean difference, 0.71; 95% CI, 0.08–1.34; *P* = 0.02), unrelated *CFH* or *CFI* rare variant carriers (mean difference, 0.98; 95% CI, 0.61–1.35; *P* < 0.001), and unrelated noncarriers (mean difference, 0.94; 95% CI, 0.72–1.16; *P* < 0.001; Fig 1B; Table S3, section B).

#### Genetic Risk Score in the Family Cohort (Familial Age-Related Macular Degeneration)

**Figure 2:**
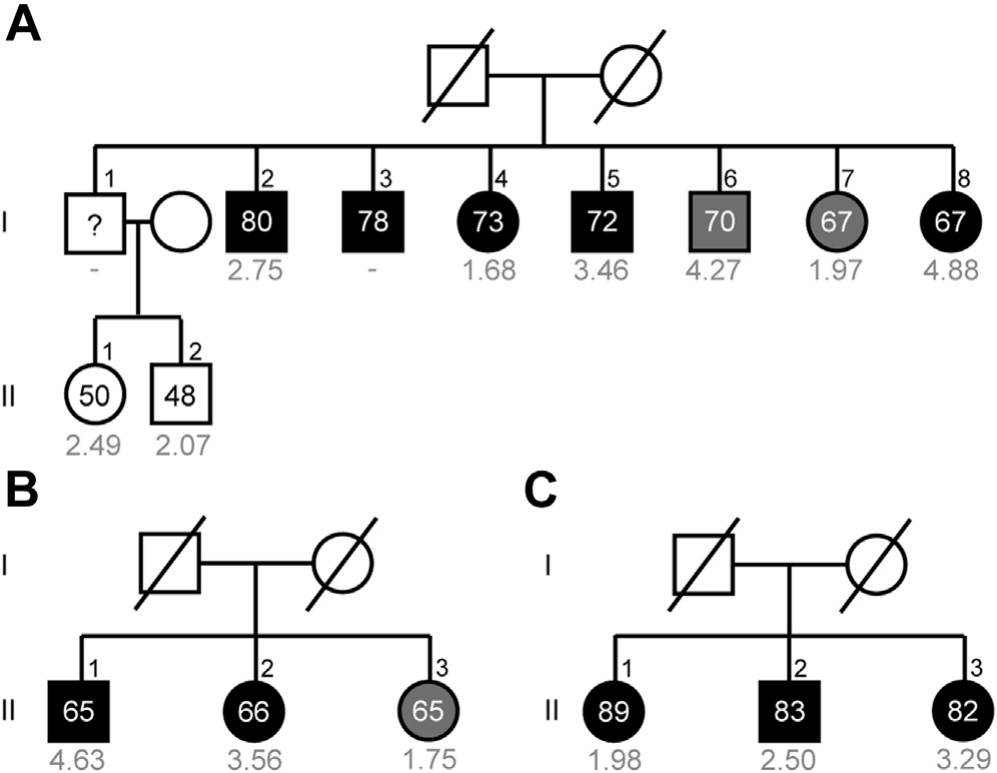
Pedigrees of 3 families with age-related macular degeneration (AMD) without rare *CFH* or *CFI* variants and a high median genetic risk score (**A**-**C**). Individuals affected by early or intermediate AMD are indicated in grey, and individuals affected by advanced AMD are indicated in black. Age at examination and the genetic risk score (in grey) are given for each individual.

**Figure 3:**
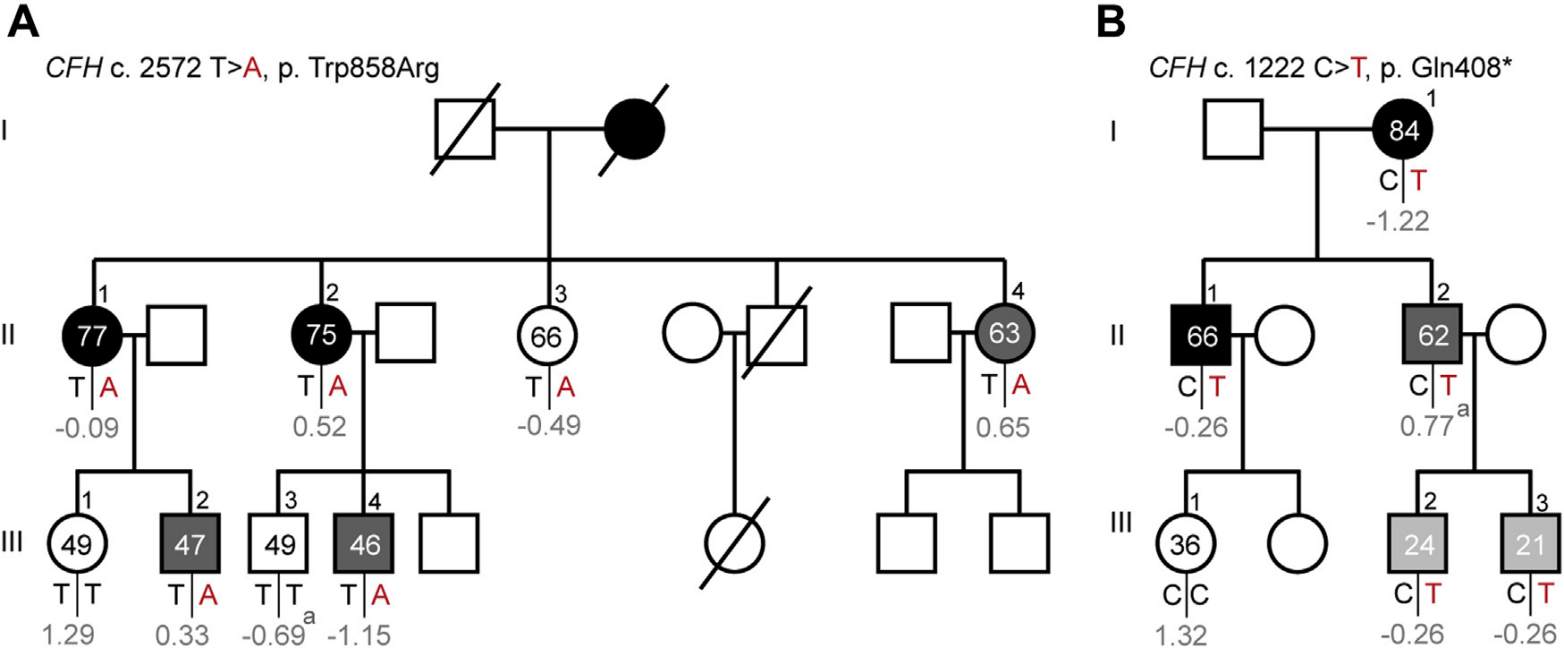
Pedigrees of 2 families with age-related macular degeneration (AMD) with rare *CFH* variants and a low median genetic risk score (**A**-**B**). Individuals affected by early or intermediate AMD are indicated in grey, and individuals affected by advanced AMD are indicated in black. Individual III.2 and III.3 from family B are affected by peripheral cuticular drusen (**B**, indicated in light grey). Age at examination and the genetic risk score (in grey) are given for each individual. A rare variant in the *CFH* gene was identified in family A, c.2572T→A, p.Trp858Arg (**A**), and in family **B**, c.1222C→T, p.Gln408∗ (**B**). Carriers of the risk allele are indicated in red. ^a^Genetic risk score of individual III.3 from family A (**A**) and individual II.2 from family B (**B**) is incomplete because they have a missing genotype in 2 of the 5 major risk alleles.

When focusing on the family cohort, we observed that carriers of rare *CFH* or *CFI* variants showed a significantly lower GRS compared with the noncarriers (mean difference, 0.71; 95% CI, 0.08–1.34; *P* = 0.02; Fig 1C; Table S3, section B). Examples of 3 families with AMD without rare *CFH* or *CFI* variants and with a high GRS and 2 families with AMD with rare *CFH* variants and with a low GRS are shown in Figures 2 and 3. Next, we analyzed GRS differences while taking into account both AMD disease stage and group category. Within the family cohort, we did not observe any differences in GRS between *CFH* or *CFI* rare variant carriers and noncarriers in individuals without AMD (mean difference, 0.47; 95% CI, –1.16 to 2.10; *P >* 0*.*99). We did observe a trend toward lower GRS in *CFH* or *CFI* rare variant carriers with early or intermediate AMD compared with noncarriers with early or intermediate AMD (mean difference, 0.64; 95% CI, –0.16 to 1.45; *P* = 0.21) and significantly lower GRS in *CFH* or *CFI* rare variant carriers with advanced AMD compared with noncarriers with advanced AMD (mean difference, 1.02; 95% CI, 0.47–1.57; *P* < 0.001; Fig 1C; Table S3, available at www.ophthalmologyscience.org, sections C-1 to C-3).

#### Genetic Risk Score in the Case-Control Cohort

Analysis of the unrelated case-control cohort revealed no differences in GRS between *CFH* or *CFI* rare variant carriers and noncarriers (mean difference, 0.05; 95% CI, –0.26 to 0.36; *P >* 0.99). Next, we analyzed GRS differences while taking into account both AMD disease stage and group category. No differences in GRS between *CFH* or *CFI* rare variant carriers and noncarriers were observed (Fig 1D; Table S3, sections C-1 to C-3).

#### Genetic Risk Score Categories in Families with Age-Related Macular Degeneration

We calculated the median GRS per family (144 families in total) and assigned each family to 1 of the 3 GRS categories (low, intermediate, or high) based on GRS tertiles derived from the case-control cohort (Table S4, available at www.ophthalmologyscience.org). In total, 77 of 144 families (53.5 %) were assigned to the high GRS category and 45 of 144 families (31.3 %) and 18 of 144 families (12.5 %) were assigned to the intermediate and low GRS categories, respectively. The families were stratified into familial AMD (at least 2 affected individuals in the family) and individuals with sporadic AMD (families with only 1 affected individual). The proportion of families in the high GRS category was higher in familial AMD (56/96 [58.3%]) compared with families with only 1 affected individual (21/48 [43.8%]).

Of the 144 families, 51 families carried rare *CFH* or *CFI* variants (35.4 %). Of the 51 families with rare *CFH* and *CFI* variants, 9 of 51 families (17.6 %) were in the low GRS category and 20 of 51 families (39.2 %) were in the high GRS category versus 9 of 93 families (9.7 %) in the low GRS category and 57 of 93 families (61.3 %) in the high GRS category among the families without rare *CFH* and *CFI* variants. In some families with familial AMD, the GRS was low and no rare *CFH* or *CFI* variants were identified (6 families). Therefore, we evaluated other genes (*C3*, *C9*, *TIMP3*, and *SLC16A8*) for the presence of rare variants in these 6 families. In 1 family, a rare *C3* variant (c.481C→T, p.Arg161Trp) was identified, and in another family, a rare *C3* variant (c.4855A→T, p.Ser1619Arg) and a rare *C9* variant (c.352C→T, p.Arg118Trp) were identified. The combined annotation-dependent depletion scores,^29^ which predict the deleteriousness of single nucleotide variants, were high (23.6, 22.3, and 23.0, respectively).

Lastly, we focused on GRS categories in familial AMD on an individual level instead of a familial level and evaluated the proportion of individuals affected by any AMD to elucidate whether a high GRS based on common variants or carriership of a rare *CFH* or *CFI* variant could contribute to AMD. In total, 307 of 553 family members were affected by any AMD. A high GRS was observed in 176 of 307 affected family members (57.3%), whereas in a smaller proportion of affected family members, an intermediate GRS (60/307 [19.5%]) or a low GRS (30/307 [9.8%]) were observed. In 41of 307 affected family members (13.4%), GRS was not available. In the affected family members with a low or intermediate GRS (n = 90), we evaluated if they carried a rare *CFH* or *CFI* variant that could contribute to AMD development. In 36 of 90 affected family members (40.0 %), a rare missense, frameshift, or splice-site variant in the *CFH* or *CFI* genes was identified. This percentage was lower in affected family members with a high GRS, which was 15.9% (28/176; *P* < 0.001, chi-square test). Furthermore, advanced AMD was observed in 20 of 28 affected family members (71.4%) with both a high GRS and a rare *CFH* or *CFI* variant, at a mean ± standard deviation age of 74.1 ± 9.5 years. This proportion was lower in the remaining affected family members (n = 279); in 144 of 279 affected family members (51.6%), we observed advanced AMD at a mean ± standard deviation age of 75.7 ± 8.4 years.

### Segregation of Rare *CFH* and *CFI* Variants in Families with Age-Related Macular Degeneration

**Figure 4:**
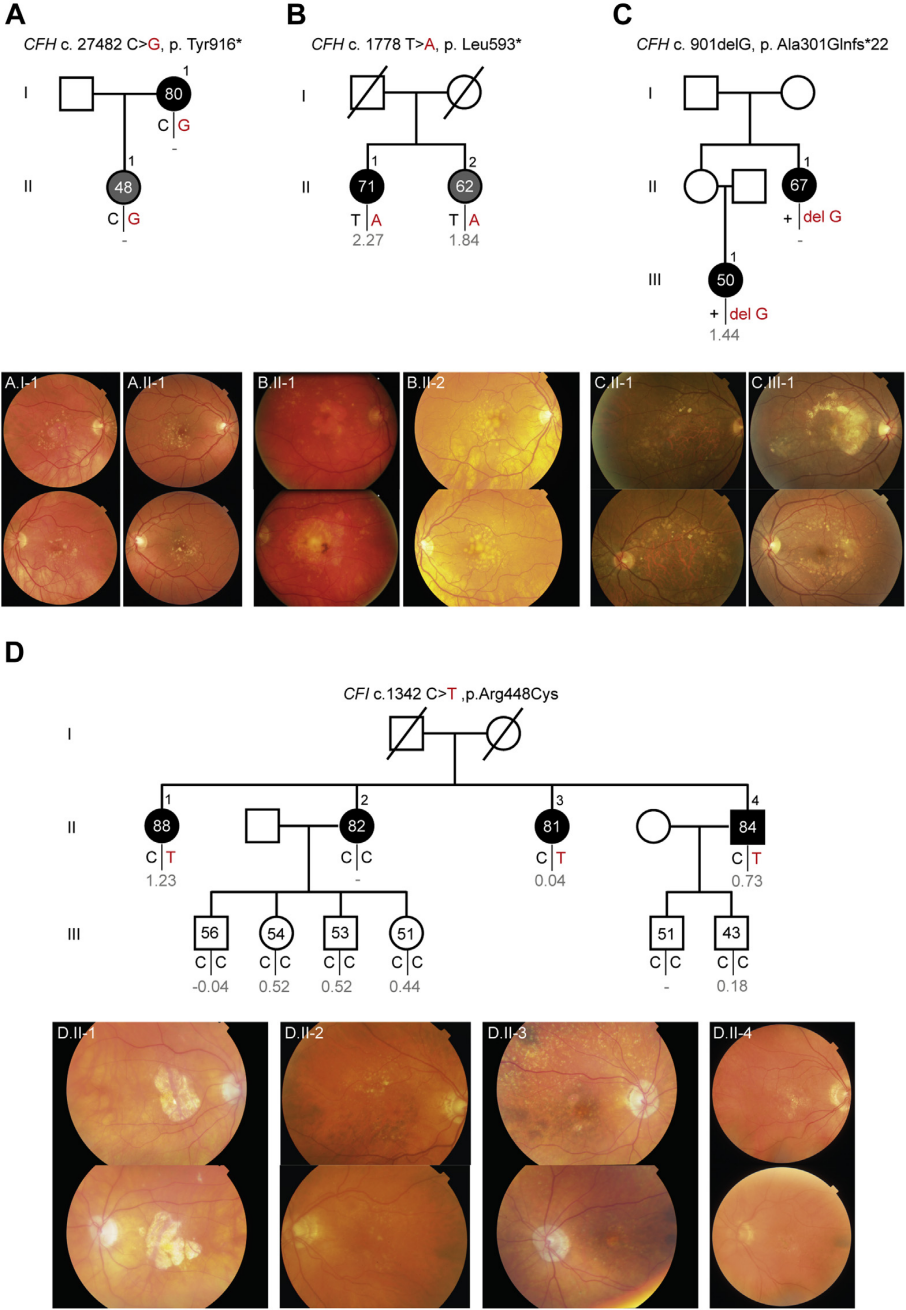
Pedigrees with accompanying color fundus photographs of 4 families with age-related macular degeneration (AMD) carrying rare *CFH* and *CFI* variants that segregate with AMD phenotype. Individuals affected by early or intermediate AMD are indicated in grey, and individuals affected by advanced AMD are indicated in black. Age at examination and the genetic risk score (in grey) are given for each individual. Carriers of the risk allele are indicated in red. **A**, Family carrying the rare *CFH* p.Tyr916∗ variant. **B**, Family carrying the rare *CFH* p.Leu593∗ variant. **C**, Family carrying the rare CFH p.Ala301Glnfs∗22 variant. **D**, Family carrying the rare *CFI* p.Arg448Cys variant.

In total, 51 of 144 families carried rare variants in the *CFH* or *CFI* genes and were included in the analysis of rare variants in families with AMD. The other families with AMD were excluded from this analysis because no rare *CFH* or *CFI* variants were identified in these families (Fig S1). In total, we identified 132 carriers of 145 rare protein-altering or splice-site variants in the *CFH* or *CFI* genes. Of the 132 carriers, 120 (90.9%) carried 1 rare *CFH* or *CFI* variant and 12 of 132 (9.1%) carried 2 rare *CFH* variants, *CFI* variants, or both. Of the unique rare variants, 28 were *CFH* variants and 11 were *CFI* variants (Fig S2, available at www.ophthalmologyscience.org). We determined whether the identified rare *CFH* or *CFI* variants in the families with AMD segregated with AMD phenotype by calculating the fraction of individuals carrying a given rare *CFH* or *CFI* variant that manifests AMD (Table 2). In this initial analysis, we included all family members. Subanalyses excluding young family members and stratification of the advanced and nonadvanced disease stages are provided in Table S5 (available at www.ophthalmologyscience.org). Rare *CFH* and *CFI* variants that were identified only once were not taken along (n = 13). Several rare variants segregated completely with AMD phenotype, whereas other rare variants showed incomplete segregation. However, among the families with incomplete segregation are several young carriers who did not show any signs of AMD yet, but could still demonstrate AMD characteristics with increasing age. Figure 4 shows 3 examples of families carrying rare *CFH* variants that segregated with AMD phenotype.

**Table 2:** Segregation of Rare *CFH* and *CFI* Variants in Families with Age-Related Macular Degeneration

Gene	Complement DNA	Protein Change	No. of Carriers	No. of Affected Carriers (Any Age-Related Macular Degeneration)/Total No. of Carriers (%)	Genetic Risk Score Carriers, Median
*CFH*	481G→T	Ala161Ser	4	1/4 (25.0%)	0.882
*CFH*	524G→A	Arg175Gln	13	11/13 (84.6%)	0.319
*CFH*	2329A→G	Ile777Val	2	1/2 (50.0%)	–0.555
*CFH*	550delA	Ile184Leufs∗32	2	2/2 (100.0%)	1.549
*CFH*	578C→T; 908G→A	Ser193Leu; Arg303Gln†	9	6/9 (66.7%)	0.885
*CFH*	607_610dupCCAA	Lys204Thrfs∗26	3	3/3 (100.0%)	0.895
*CFH*	764G→A	Gly255Glu†	3	2/3 (66.7%)	0.494
*CFH*	901delG	Ala301Glnfs∗22	2	2/2 (100.0%)	1.436
*CFH*	1198C→A	Gln400Lys	4	1/4 (25.0%)	0.043
*CFH*	1215G→T	Lys405Asn	2	1/2 (50.0%)	1.464
*CFH*	1222C→T	Gln408∗†	6	6/6 (100.0%)	–0.302
*CFH*	1697-17_1697-8del	—	3	1/3 (33.3%)	–1.019
*CFH*	1778T→A	Leu593∗	2	2/2 (100.0%)	2.054
*CFH*	2572T→A	Trp858Arg	6	5/6 (83.3%)	0.117
*CFH*	2596+8G→T	—	2	1/2 (50.0%)	2.93
*CFH*	2748C→G	Tyr916∗	2	2/2 (100.0%)	N/A
*CFH*	2850G→T	Gln950His†	13	4/13 (30.8%)	0.904
*CFH*	2867C→T	Thr956Met†	2	2/2 (100.0%)	2.862
*CFH*	3234G→T	Arg1078Ser	3	2/3 (66.7%)	1.12
*CFI*	1657C→T	Pro553Ser†	4	3/4 (75.0%)	1.63
*CFI*	1342C→T	Arg448Cys†	4	4/4 (100.0%)	0.982
*CFI*	1016G→A	Arg339Gln	3	2/3 (66.7%)	2.169
*CFI*	392T→G	Leu131Arg	5	3/5 (60.0%)	1.145
*CFI*	563G→C	Gly188Ala	5	3/5 (60.0%)	1.778
*CFI*	355G→A	Gly119Arg†	18	14/18 (77.8%)	2.784

– = not applicable.

Rare *CFH* and *CFI* variants were identified in at least 2 family members. The fraction of individuals carrying a given rare *CFH* or *CFI* variant that manifests age-related macular degeneration was determined.

† Variants that were identified in multiple families.

In family A, the 80-year-old mother and her 47-year-old daughter both carried a heterozygous nonsense mutation in the *CFH* gene (p.Tyr916∗). A large area of central GA was found in the right eye of the mother. Color fundus photography of the daughter showed AMD characteristics similar to the intermediate AMD stage at a remarkably young age. Another heterozygous nonsense mutation in the *CFH* gene (p.Leu593∗) was identified in family B. Both siblings carried the mutation and were affected by AMD. The 62-year-old woman (II.2) harbored large confluent macular drusen in both eyes without any signs of advanced AMD, whereas her 71-year-old sister (II.1) harbored a large area of central GA in the right eye and a fibrous scar secondary to a CNV together with atrophic spots in the macula in the left eye. In addition to the rare variant, both siblings showed a relative high GRS (GRS, 2.27 in II.2 and 1.84 in II.4). In the 2 individuals from family C, a heterozygous frameshift variant in the *CFH* gene (p.Ala301Glnfs∗22) was identified. A large area of GA was visible in the central macula of both eyes of the 67-year-old woman. The phenotype of the other individual in this family (III.1) was even more severe. This 50-year-old woman received a diagnosis of early onset drusen maculopathy at 44 years of age and by 50 years of age she demonstrated a CNV in the right eye, whereas the left eye had not progressed to an advanced disease stage yet. Nine years later, a CNV with submacular hemorrhage developed in her left eye, with consequent visual loss. In family D, a heterozygous missense variant in the *CFI* gene (p.Arg448Cys) was identified in 3 individuals of this family. All 3 individuals carrying this rare variant demonstrated GA of variable sizes in both eyes. The fourth sibling of this family (II.2) did not carry the rare *CFI* p.Arg448Cys variant, but also was affected by advanced AMD. However, instead of GA, this individual demonstrated a CNV in the right eye. Although the GRS of this individual was missing, she carried the *CFH* rs1061170 (p.Tyr402His) risk allele homozygously, whereas she did not carry the *ARMS2* rs10490924 (p.Ala69Ser) risk allele. The complete overview of rare *CFH* and *CFI* variants identified in the 51 families with AMD, including family structure and subanalysis of the segregation of rare *CFH* and *CFI* variants, is depicted in Table S5.

**Figure 5:**
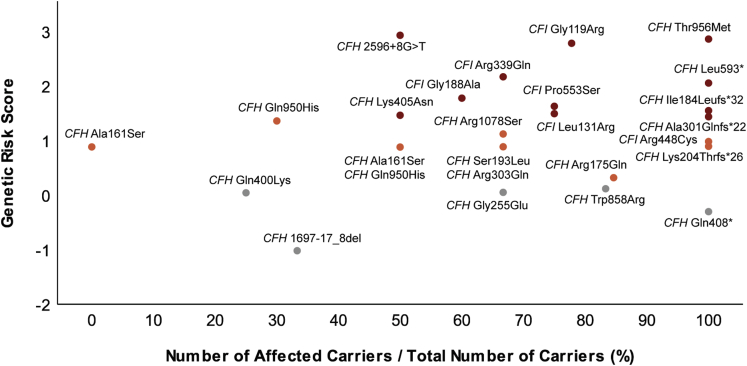
Graph showing the segregation patterns and genetic risk scores (GRSs) of carriers of rare *CFH* and *CFI* variants that manifest age-related macular degeneration (AMD). The y-axis represents the median genetic risk score per variant, and the x-axis represents the number of affected carriers divided by the total number of carriers (percentage). The variants are color coded according to the GRS category: grey dots correspond to the low GRS category (≤0.220), orange dots correspond to the intermediate GRS category (0.221–1.407), and red dots correspond to the high GRS category (≥1.408).

Next, we evaluated the segregation pattern and GRS (median) per rare variant (Fig 5). Two rare *CFH* variants (*CFH* p.Trp858Arg and *CFH* p.Gln408∗) stand out because the genetic risk based on common variants is low (GRS, 0.12 and –0.30, respectively), whereas the fraction of carriers of these variants that manifests AMD is high (83.3% and 100.0%, respectively).

## Discussion

In this study, we provided insight into the contribution of common and rare genetic risk variants in families with AMD. We observed a higher genetic load based on common variants in individuals from families with AMD. In 57.3% (176/307) of the affected family members in the familial AMD cohort, a high GRS contributed to AMD development, whereas rare *CFH* and *CFI* variants contributed to AMD development in 40.0% (36/90) of the affected family members with a low and intermediate GRS. Interestingly, carriers of rare *CFH* and *CFI* variants in the family cohort showed a lower GRS compared with the *CFH* and *CFI* noncarriers (Fig 1). In addition, we determined the segregation patterns of rare *CFH* and *CFI* variants in families with AMD and identified several rare *CFH* and *CFI* variants that showed a high segregation rate with AMD phenotype (Table 2).

With this study, we showed that the identification of rare *CFH* and *CFI* variants in addition to common variants is important because in some families with AMD, the genetic risk is mainly determined by a high genetic load based on common genetic variants (Fig 2), whereas in other families with AMD, the GRS is low and a rare *CFH* or *CFI* variant segregates with the disease and contribute to AMD development (Fig 3). Some families have an even higher genetic burden. They demonstrate a high GRS based on common variants, and in addition, a highly penetrant rare *CFH* or *CFI* variant runs in the family. Results of the current study are in line with those of previous studies. In a large family study by Sobrin et al,^12^ the authors reported that common variants explained the disease in most of the families and hypothesized that in the families whose disease could not be explained by common variants, more penetrant rare variants might contribute to the disease. This hypothesis also was stated in other studies; however, they included only a small number of families (≤22 families).^11^^,^^13^^,^^14^^,^^18^ In the current study, where we included a large number of families (n = 144), we confirmed this hypothesis by identifying families with AMD with a low GRS and a rare *CFH* or *CFI* variant that segregated completely or almost completely with the disease.

A small number of families with familial AMD demonstrated a low GRS and did not carry rare *CFH* or *CFI* variants (6 families). In 1 family, a rare *C3* variant was identified (c.481C→T, p.Arg161Trp), and in another family, a rare *C3* variant (c.4855A→C, p.Ser1619Arg) and a rare *C9* variant (c.352C→T, p.Arg118Trp) were identified. These variants could contribute to the development of AMD in these families because they were predicted to be damaging based on the combined annotation-dependent depletion prediction score (>20 for all 3 variants). In the remaining 4 families, genetic variants in other genes may be involved. Environmental factors could also play a role, but these factors were not taken into account in the current study.

Other rare *CFH* and *CFI* variants identified in the current study did not segregate (completely) with the disease. It must be noted that we identified multiple young rare *CFH* and *CFI* variant carriers in the families. It is possible that they will demonstrate AMD characteristics in the future, and therefore, we performed a subanalysis by excluding individuals younger than 65 years. These young individuals should be invited for follow-up studies to determine their disease status after they reach 65 years of age. One also should take into account the pathogenicity of the rare variant. Although functional studies have been performed for several rare *CFH* and *CFI* variants that were identified in AMD, for many of them, the functional effect remains unknown (Appendix A of De Jong et al^30^). For 19 of 40 unique rare *CFH* and *CFI* variants identified in families with AMD in our study, functional studies were performed. Most of those variants resulted in reduced protein levels or reduced function. For the remaining 21 rare *CFH* variants, we currently are measuring factor H levels and complement activation markers to determine their functional effect (De Jong et al, Human Molecular Genetics, in press). Furthermore, the multifactorial cause of AMD makes the story even more complex because other risk factors, such as lifestyle, are also involved in the pathogenesis of AMD.

The availability of extensive genotyping data and the large number of families with AMD in the European Genetic Database is a major strength of this study. It allowed us to identify a relatively large number of rare *CFH* and *CFI* variants in families with AMD. Part of the rare *CFH* and *CFI* variants identified within the European Genetic Database were identified by whole exome sequencing and described before,^16^, ^17^, ^18^^,^^20^^,^^31^ and recently, we identified additional *CFH* and *CFI* rare variant carriers by single-molecule molecular inversion probes.^10^ Furthermore, new family members and several new rare *CFH* and *CFI* variant carriers were included from the outpatient clinic of the Radboud University Medical Center. In particular, 2 variants in the *CFH* gene (p.Tyr916∗ and p.Leu593∗) are of interest because they caused a severe AMD phenotype at a relatively young age in several patients in the current study (Fig 4) and have not yet been reported in patients with AMD. In the literature, clustering of low-frequency variants in the N-terminal complement control protein (CCP) domains 1 to 4 of factor H was reported in patients with AMD.^32^ The variants Leu593∗ and Tyr916∗ are located in CCP domains 10 and 16, respectively. Fifty percent of the unique rare *CFH* variants identified in families with AMD in the current study are located in the first 7 CCP domains of factor H, whereas the other 50% of the rare *CFH* variants are spread across the other CCP domains of FH (Fig S2), indicating that rare variants in the complete *CFH* gene are relevant with respect to familial AMD.

### Study Limitations

The current study has several limitations in. Despite the high number of identified rare *CFH* and *CFI* variants, we were not able to determine the segregation patterns for all these variants because for some variants, only 1 carrier was identified. Second, the families included multiple young carriers who had not yet reached the age of onset of AMD. In future studies, additional family members of rare variant carriers should be collected to better understand the segregation patterns of specific rare variants. In the current study, we calculated the fraction of individuals carrying a given rare *CFH* or *CFI* variant that manifests AMD. Because in the European Genetic Database specific subgroups, such as families with rare variants, are enriched, these numbers may not be representative for the general AMD population. Furthermore, in families with both a high GRS and a rare *CFH* or *CFI* variant that segregates with the disease, it is challenging to determine which one is the main driver of the disease in that particular family; however, it is likely that both contribute. In families with both a low GRS and no rare *CFH* and *CFI* variants, other rare genetic variants in complement genes or genes in other pathways could contribute to AMD development that were not evaluated in detail in the current study. Finally, in some individuals, extensive genotyping data were not available. Despite these limitations, this study performs a detailed GRS analysis in *CFH* and *CFI* rare variant carriers and noncarriers in a large cohort of families with AMD and an unrelated case-control cohort.

In conclusion, we demonstrated that individuals from families with AMD are at high risk of developing AMD because they often have a high GRS based on common variants, carry a rare *CFH* or *CFI* variant that segregates with AMD phenotype, or both. Assessing a GRS and sequencing of the *CFH* and *CFI* genes is important to generate a more complete genetic picture, which is valuable for family counseling and for developing personalized medicine approaches. Importantly, carriers of rare *CFH* and *CFI* variants are eligible candidates for ongoing complement trials for AMD targeting specific genotypes (https://www.clinicaltrialsregister.eu/ctr-search/trial/2019–003421–22/GB, https://clinicaltrials.gov/ct2/show/study/NCT04246866) and for potential future treatments. Additional functional studies are essential to determine the functional effect and clinical relevance of rare *CFH* and *CFI* variants.

## References

[1] Colijn J.M., Buitendijk G.H.S., Prokofyeva E. (2017). Prevalence of age-related macular degeneration in Europe: the past and the future. Ophthalmology.

[2] Flaxman S.R., Bourne R.R.A., Resnikoff S. (2017). Global causes of blindness and distance vision impairment 1990–2020: a systematic review and meta-analysis. Lancet Glob Health.

[3] Meyers S.M., Greene T., Gutman F.A. (1995). A twin study of age-related macular degeneration. Am J Ophthalmol.

[4] Seddon J.M., Cote J., Page W.F. (2005). The US twin study of age-related macular degeneration: relative roles of genetic and environmental influences. Arch Ophthalmol.

[5] Klaver C.C., Wolfs R.C., Assink J.J. (1998). Genetic risk of age-related maculopathy. Population-based familial aggregation study. Arch Ophthalmol.

[6] Seddon J.M., Ajani U.A., Mitchell B.D. (1997). Familial aggregation of age-related maculopathy. Am J Ophthalmol.

[7] Fritsche L.G., Igl W., Bailey J.N. (2016). A large genome-wide association study of age-related macular degeneration highlights contributions of rare and common variants. Nat Genet.

[8] Geerlings M.J., de Jong E.K., den Hollander A.I. (2017). The complement system in age-related macular degeneration: a review of rare genetic variants and implications for personalized treatment. Mol Immunol.

[9] Colijn J.M., Meester-Smoor M., Verzijden T. (2021). Genetic risk, lifestyle, and age-related macular degeneration in Europe: the EYE-RISK Consortium. Ophthalmology.

[10] de Breuk A., Acar I.E., Kersten E. (2021). Development of a genotype assay for age-related macular degeneration: the EYE-RISK Consortium. Ophthalmology.

[11] Hoffman J.D., Cooke Bailey J.N., D’Aoust L. (2014). Rare complement factor H variant associated with age-related macular degeneration in the Amish. Invest Ophthalmol Vis Sci.

[12] Sobrin L., Maller J.B., Neale B.M. (2010). Genetic profile for five common variants associated with age-related macular degeneration in densely affected families: a novel analytic approach. Eur J Hum Genet.

[13] Wagner E.K., Raychaudhuri S., Villalonga M.B. (2016). Mapping rare, deleterious mutations in factor H: association with early onset, drusen burden, and lower antigenic levels in familial AMD. Sci Rep.

[14] Yu Y., Triebwasser M.P., Wong E.K. (2014). Whole-exome sequencing identifies rare, functional CFH variants in families with macular degeneration. Hum Mol Genet.

[15] Saksens N.T., Lechanteur Y.T., Verbakel S.K. (2016). Analysis of risk alleles and complement activation levels in familial and non-familial age-related macular degeneration. PLoS One.

[16] Boon C.J., Klevering B.J., Hoyng C.B. (2008). Basal laminar drusen caused by compound heterozygous variants in the CFH gene. Am J Hum Genet.

[17] Duvvari M.R., van de Ven J.P., Geerlings M.J. (2016). Whole exome sequencing in patients with the cuticular drusen subtype of age-related macular degeneration. PLoS One.

[18] Saksens N.T., Geerlings M.J., Bakker B. (2016). Rare genetic variants associated with development of age-related macular degeneration. JAMA Ophthalmol.

[19] Taylor R.L., Poulter J.A., Downes S.M. (2019). Loss-of-function mutations in the CFH gene affecting alternatively encoded factor H-like 1 protein cause dominant early-onset macular drusen. Ophthalmology.

[20] van de Ven J.P., Boon C.J., Fauser S. (2012). Clinical evaluation of 3 families with basal laminar drusen caused by novel mutations in the complement factor H gene. Arch Ophthalmol.

[21] Ristau T., Ersoy L., Lechanteur Y. (2014). Allergy is a protective factor against age-related macular degeneration. Invest Ophthalmol Vis Sci.

[22] Bird A.C., Bressler N.M., Bressler S.B. (1995). An international classification and grading system for age-related maculopathy and age-related macular degeneration. The International ARM Epidemiological Study Group. Surv Ophthalmol.

[23] Klein R., Davis M.D., Magli Y.L. (1991). The Wisconsin age-related maculopathy grading system. Ophthalmology.

[24] van Leeuwen R., Chakravarthy U., Vingerling J.R. (2003). Grading of age-related maculopathy for epidemiological studies: is digital imaging as good as 35-mm film?. Ophthalmology.

[25] Corominas J., Colijn J.M., Geerlings M.J. (2018). Whole-exome sequencing in age-related macular degeneration identifies rare variants in COL8A1, a component of Bruch’s membrane. Ophthalmology.

[26] Duvvari M.R., Saksens N.T., van de Ven J.P. (2015). Analysis of rare variants in the CFH gene in patients with the cuticular drusen subtype of age-related macular degeneration. Mol Vis.

[27] Geerlings M.J., Kremlitzka M., Bakker B. (2017). The functional effect of rare variants in complement genes on C3b degradation in patients with age-related macular degeneration. JAMA Ophthalmol.

[28] van de Ven J.P., Nilsson S.C., Tan P.L. (2013). A functional variant in the CFI gene confers a high risk of age-related macular degeneration. Nat Genet.

[29] Kircher M., Witten D.M., Jain P. (2014). A general framework for estimating the relative pathogenicity of human genetic variants. Nat Genet.

[30] de Jong S., Gagliardi G., Garanto A. (2021). Implications of genetic variation in the complement system in age-related macular degeneration. Prog Retin Eye Res.

[31] Kersten E., Geerlings M.J., den Hollander A.I. (2017). Phenotype characteristics of patients with age-related macular degeneration carrying a rare variant in the complement factor H gene. JAMA Ophthalmol.

[32] Geerlings M.J., Volokhina E.B., de Jong E.K. (2018). Genotype-phenotype correlations of low-frequency variants in the complement system in renal disease and age-related macular degeneration. Clin Genet.

