# Supplementary material for: Genetic Risk in Families with Age-Related Macular Degeneration
Source: Ophthalmol Sci. 2021 Dec 6;1(4):100087. doi: 10.1016/j.xops.2021.100087 (PMC9562327; doi:10.1016/j.xops.2021.100087)
Supplement: Fig S1 [file mmc1.pdf]

Supplementary Figure 1. Flow chart for selection of study cohorts

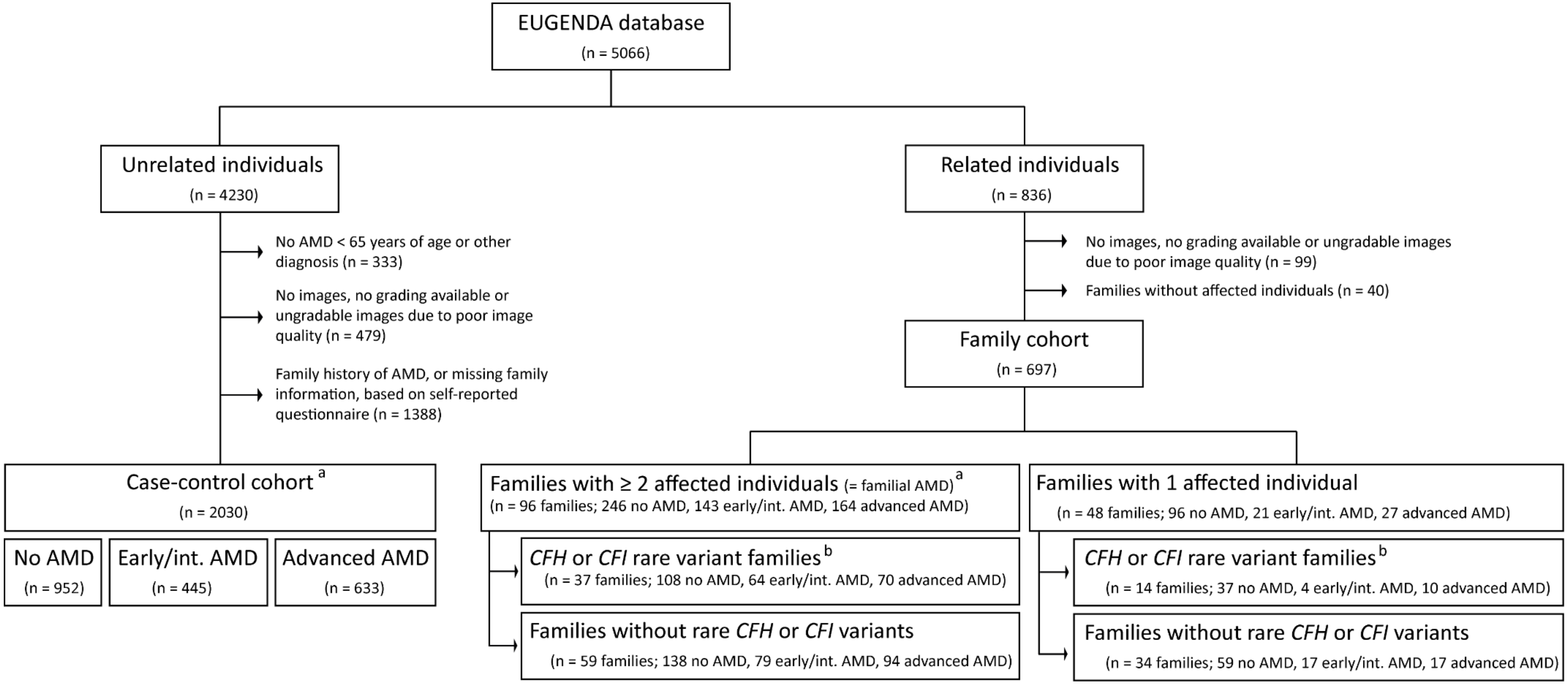

Flow chart of the selection of the study cohorts. [a] Cohorts included in GRS analysis. [b] Cohorts included in rare variant analysis. AMD = age-related macular degeneration; EUGENDA = European Genetic Database; CFH = complement factor H; CFI = complement factor I; early/int. AMD = early/intermediate AMD.
