# Supplementary figures and images for: Genetic Risk in Families with Age-Related Macular Degeneration
Source: Ophthalmol Sci. 2021 Dec 6;1(4):100087. doi: 10.1016/j.xops.2021.100087 (PMC9562327; doi:10.1016/j.xops.2021.100087)

Supplementary Figure 2. Overview of identified rare *CFH* and *CFI* variants in AMD families

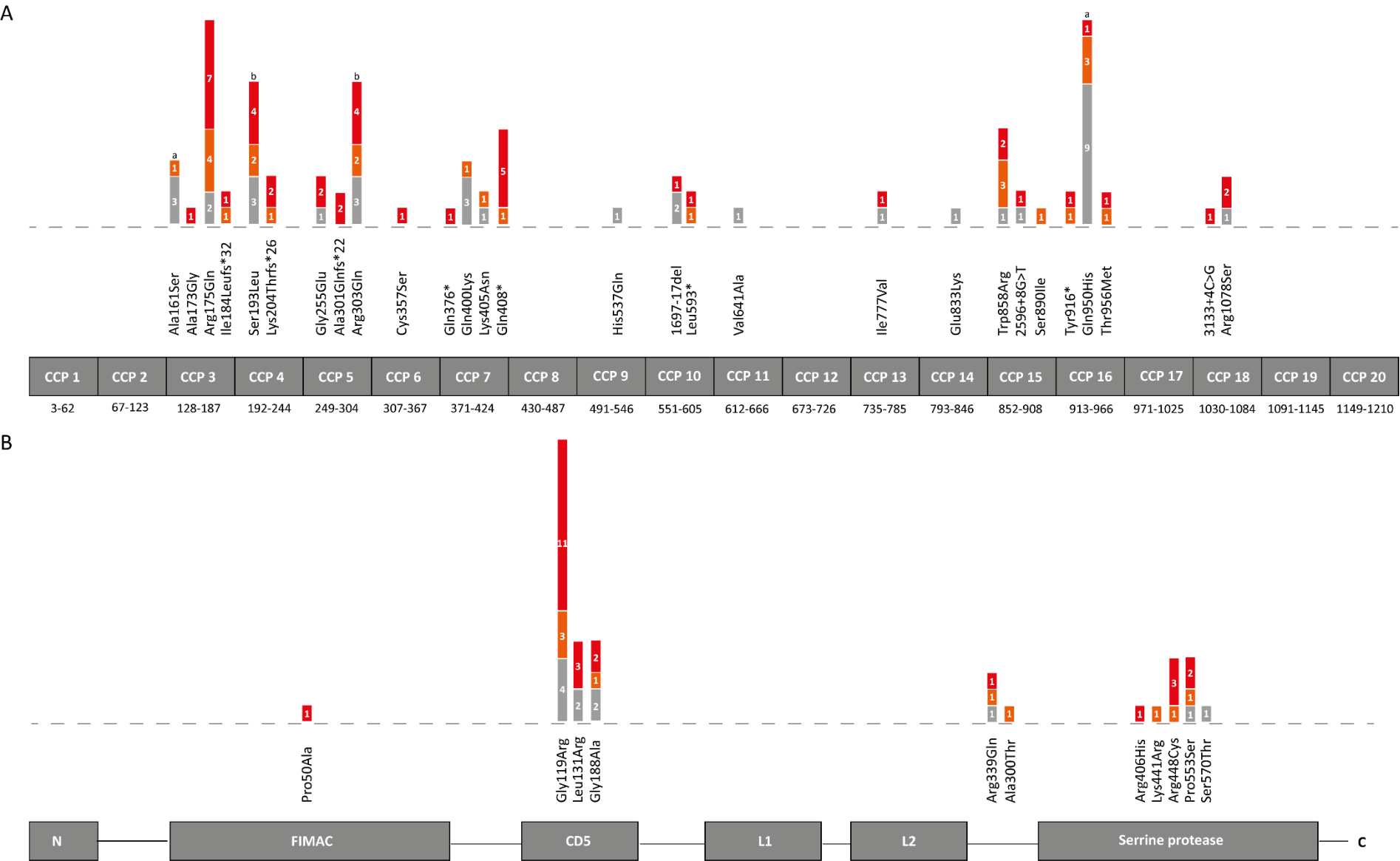

Supplement: Fig S2 [file mmc2.pdf]
