# Supplementary material for: Genetic Risk in Families with Age-Related Macular Degeneration
Source: Ophthalmol Sci. 2021 Dec 6;1(4):100087. doi: 10.1016/j.xops.2021.100087 (PMC9562327; doi:10.1016/j.xops.2021.100087)
Supplement: Table S1 [file mmc3.pdf]

Supplementary Table 1. Genetic risk score in familial AMD and unrelated individuals

		N	GRS, mean	SD	GRS, mean, estimates [a]	SE	Age, mean (SD)
Group category	**Familial *CFH* or *CFI* rare variant carriers**						
	No AMD	4	0.566	1.563	0.566	0.593	71 (8)
	Early/intermediate AMD	18	1.225	1.222	1.225	0.280	59 (15)
	Advanced AMD	46	1.366	1.309	1.366	0.175	74 (10)
	Subtotal	*68*	*1.281*	*1.294*	*1.052*	*0.226*	*70 (13)*
	**Familial noncarriers**						
	No AMD	51	1.035	1.050	1.035	0.166	70 (4)
	Early/intermediate AMD	95	1.867	1.256	1.867	0.122	67 (10)
	Advanced AMD	107	2.385	1.180	2.385	0.115	76 (8)
	Subtotal	*253*	*1.918*	*1.282*	*1.762*	*0.079*	*71 (9)*
	**Unrelated *CFH* or *CFI* rare variant carriers**						
	No AMD	36	-0.099	1.178	-0.099	0.198	71 (5)
	Early/intermediate AMD	28	0.739	1.047	0.739	0.224	73 (10)
	Advanced AMD	47	1.708	1.390	1.708	0.173	76 (9)
	Subtotal	*111*	*0.878*	*1.460*	*0.783*	*0.115*	*74 (9)*
	**Unrelated noncarriers**						
	No AMD	628	0.187	1.110	0.187	0.047	73 (7)
	Early/intermediate AMD	394	0.612	1.244	0.612	0.060	73 (10)
	Advanced AMD	557	1.700	1.202	1.700	0.050	77 (9)
	Subtotal	*1579*	*0.827*	*1.352*	*0.833*	*0.030*	*74 (9)*
AMD disease stage	No AMD	719	0.235	1.133	0.422	0.162	73 (7)
	Early/intermediate AMD	535	0.862	1.323	1.111	0.096	72 (10)
	Advanced AMD	757	1.777	1.243	1.790	0.069	76 (9)

Mean genetic risk score in CFH and CFI rare variant carriers in familial AMD and unrelated individuals. [a] estimated marginal means. N = number of individuals in each category; Italic numbers indicate the subtotals of four group categories (familial CFH or CFI rare variant carriers, familial noncarriers, unrelated CFH or CFI rare variant carriers, unrelated noncarriers) and the three AMD disease stages (no AMD, early/intermediate AMD, advanced AMD). GRS = genetic risk score; SD = standard deviation; SE = standard error; AMD = age-related macular degeneration; CFH = complement factor H; CFI = complement factor I.
