# Supplementary material for: Genetic Risk in Families with Age-Related Macular Degeneration
Source: Ophthalmol Sci. 2021 Dec 6;1(4):100087. doi: 10.1016/j.xops.2021.100087 (PMC9562327; doi:10.1016/j.xops.2021.100087)
Supplement: Table S2 [file mmc4.pdf]

Supplementary Table 2. Main effects two-way analysis of variance

**Source**	***df***	**MS**	***F***	**P-value**	**Partial Eta Squared**
Corrected model	11	100.020	71.118	< 0.001	0.281
Intercept	1	386.074	274.514	< 0.001	0.121
Group category	3	58.160	41.354	< 0.001	0.058
AMD disease stage	2	54.090	38.460	< 0.001	0.037
Group category*AMD disease stage	6	4.321	3.072	0.005	0.009
Error	1999	1.406			
Total	2011				
Corrected total	2010				

Results of the main effects of the genetic risk score comparison, analyzed by a two-way analysis of variance (ANOVA). P-values < 0.05 are considered statistically significant. *Df* = degrees of freedom, MS = mean squares, *F* = F-test, Partial eta squared represents the effect size.
