# Supplementary material for: Genetic Risk in Families with Age-Related Macular Degeneration
Source: Ophthalmol Sci. 2021 Dec 6;1(4):100087. doi: 10.1016/j.xops.2021.100087 (PMC9562327; doi:10.1016/j.xops.2021.100087)
Supplement: Table S3 [file mmc5.pdf]

Supplementary Table 3. Pairwise comparisons two-way analysis of variance

Section	Level I	Level II	Level III	GRS difference II-III (95% CI)	*p*[a]
A	N/A	Advanced AMD	Early/intermediate AMD	0.679 (0.396-0.962)	< 0.001***
	N/A	Advanced AMD	No AMD	1.367 (0.945-1.790)	< 0.001***
	N/A	Early/intermediate AMD	No AMD	0.718 (0.145-1.292)	0.001**
B	N/A	Familial noncarriers	Familial *CFH* or *CFI* rare variant carriers	0.710 (0.078-1.343)	0.02 *
	N/A	Familial noncarriers	Unrelated *CFH* or *CFI* rare variant carriers	0.980 (0.612-1.348)	< 0.001***
	N/A	Familial noncarriers	Unrelated noncarriers	0.936 (0.715-1.157)	< 0.001***
	N/A	Familial *CFH* or *CFI* rare variant carriers	Unrelated *CFH* or *CFI* rare variant carriers	0.269 (-0.401-0.939)	> 0.99
	N/A	Familial *CFH* or *CFI* rare variant carriers	Unrelated noncarriers	0.219 (-0.384-0.822)	> 0.99
	N/A	Unrelated noncarriers	Unrelated *CFH* or *CFI* rare variant carriers	0.050 (-0.264-0.364)	> 0.99
C-1	No AMD	Familial noncarriers	Familial *CFH* or *CFI* rare variant carriers	0.470 (-1.157-2.096)	> 0.99
		Familial noncarriers	Unrelated *CFH* or *CFI* rare variant carriers	1.134 (0.452-1.816)	< 0.001***
		Familial noncarriers	Unrelated noncarriers	0.849 (0.393-1.305)	< 0.001***
		Familial *CFH* or *CFI* rare variant carriers	Unrelated *CFH* or *CFI* rare variant carriers	0.665 (-0.986-2.315)	> 0.99
		Familial *CFH* or *CFI* rare variant carriers	Unrelated noncarriers	0.379 (-1.192-1.950)	> 0.99
		Unrelated noncarriers	Unrelated *CFH* or *CFI* rare variant carriers	0.285 (-0.251-0.822)	0.96
C-2	Early/intermediate AMD	Familial noncarriers	Familial *CFH* or *CFI* rare variant carriers	0.642 (-0.163-1.447)	0.21
		Familial noncarriers	Unrelated *CFH* or *CFI* rare variant carriers	1.128 (0.455-1.802)	< 0.001***
		Familial noncarriers	Unrelated noncarriers	1.255 (0.897-1.613)	< 0.001***
		Familial *CFH* or *CFI* rare variant carriers	Unrelated *CFH* or *CFI* rare variant carriers	0.486 (-0.460-1.432)	> 0.99
		Familial *CFH* or *CFI* rare variant carriers	Unrelated noncarriers	0.612 (-0.143-1.367)	0.19
		Unrelated noncarriers	Unrelated *CFH* or *CFI* rare variant carriers	-0.126 (-0.739-0.486)	> 0.99
C-3	Advanced AMD	Familial noncarriers	Familial *CFH* or *CFI* rare variant carriers	1.019 (0.467-1.571)	< 0.001***
		Familial noncarriers	Unrelated *CFH* or *CFI* rare variant carriers	0.676 (0.128-1.224)	0.007**
		Familial noncarriers	Unrelated noncarriers	0.685 (0.354-1.016)	< 0.001***
		Familial *CFH* or *CFI* rare variant carriers	Unrelated *CFH* or *CFI* rare variant carriers	-0.343 (-0.993-0.307)	0.98
		Familial *CFH* or *CFI* rare variant carriers	Unrelated noncarriers	-0.334 (-0.815-0.146)	0.40
		Unrelated noncarriers	Unrelated *CFH* or *CFI* rare variant carriers	-0.009 (-0.484-0.467)	> 0.99

Results of the genetic risk score comparison analyzed by a two-way analysis of variance (ANOVA), pairwise comparisons. Section A: pairwise comparison of AMD disease stages. Section B: pairwise comparisons of group categories. Sections C-1 to C-3: pairwise comparisons of group category stratified by AMD disease stage. [a] For the pairwise comparisons a Bonferroni correction for multiple testing was applied. * $P < 0.05$, ** $P < 0.01$,*** $P < 0.001$. *Df* = degrees of freedom, MS = mean squares, *F* = F-test, *p* = significance level α = 0.05, effect size presented as partial η^2, CI = confidence interval
