# Supplementary material for: Genetic Risk in Families with Age-Related Macular Degeneration
Source: Ophthalmol Sci. 2021 Dec 6;1(4):100087. doi: 10.1016/j.xops.2021.100087 (PMC9562327; doi:10.1016/j.xops.2021.100087)
Supplement: Table S4 [file mmc6.pdf]

Supplementary Table 4. Genetic risk score categories of AMD families

	**Families with ≥ 2 affected individuals (familial AMD) (n = 96 families)**	**Families with 1 affected individual (n = 48 families)**	***Subtotal***
**Families with rare *CFH* or *CFI* variants (n = 51 families)**			
Low GRS category	6/37 (16.2 %)	3/14 (21.4 %)	*9/51 (17.6 %)*
Intermediate GRS category	14/37 (37.8 %)	6/14 (42.9 %)	*20/51 (39.2 %)*
High GRS category	15/37 (40.5 %)	5/14 (35.7 %)	*20/51 (39.2 %)*
N/A	2/37 (5.4 %)	NA	*2/51 (3.9 %)*
**Families without rare *CFH* or *CFI* variants (n = 93 families)**			
Low GRS category	6/59 (10.2 %)	3/34 (8.8 %)	*9/93 (9.7 %)*
Intermediate GRS category	12/59 (20.3 %)	13/34 (38.2 %)	*25/93 (26.9 %)*
High GRS category	41/59 (69.5 %)	16/34 (47.1 %)	*57/93 (61.3 %)*
N/A	NA	2/34 (5.9 %)	*2/93 (2.2 %)*
Subtotal			
Low GRS category	*12/96 (12.5 %)*	*6/48 (12.5 %)*	*18/144 (12.5 %)*
Intermediate GRS category	*26/96 (27.1 %)*	*19/48 (39.6 %)*	*45/144 (31.3 %)*
High GRS category	*56/96 (58.3 %)*	*21/48 (43.8 %)*	*77/144 (53.5 %)*
N/A	*2/96 (2.1 %)*	*2/48 (4.2 %)*	*4/144 (2.8 %)*

GRS categories within the 144 families. Median GRS per family were calculated and assigned to one of the three GRS categories: low (GRS ≤ 0.220), intermediate (GRS 0.221 - 1.407), high (GRS ≥ 1.408). In four families GRS were not available. Subtotals are indicated in italic. AMD = age-related macular degeneration; GRS = genetic risk score; CFH = complement factor H; CFI = complement factor I; N/A = not available; NA = not applicable.
