# Supplementary material for: Genetic Risk in Families with Age-Related Macular Degeneration
Source: Ophthalmol Sci. 2021 Dec 6;1(4):100087. doi: 10.1016/j.xops.2021.100087 (PMC9562327; doi:10.1016/j.xops.2021.100087)
Supplement: Table S5 [file mmc7.pdf]

Supplementary Table 5. Family structure and segregation of rare *CFH* and *CFI* variants in AMD families

Variant	No. of carriers	No. of affected carriers (any AMD)/total no. of carriers (%)	No. of affected carriers (late AMD)/total no. of carriers (%)	No. of affected carriers (any AMD)/total no. of carriers (%) [age 65 threshold]	No. of affected carriers (late AMD)/total no. of carriers (%) [age 65 threshold]	GRS carriers, median	Family structure
CFH c.481G>T, p.Ala161Ser	4	1/4 (25.0 %)	0/4 (0.0 %)	1/2 (50.0 %)	0/2 (0.0 %)	0.882	Three generations; one affected and five unaffected[a]
CFH c.518C>G, p.Ala173Ser	1	ND	ND	ND	ND	2.736	Two generations; one affected and two unaffected[b]
CFH c.524G>A, p.Arg175Gln	13	11/13 (84.6 %)	7/13 (53.8 %)	11/11 (100.0 %)	7/11 (63.3 %)	0.319	Three generations; fourteen affected and twelve unaffected
CFH c.2329A>G, p.Ile777Val	2	1/2 (50 %)	1/2 (50 %)	ND	ND	-0.555	Two generations; three affected and four unaffected
CFH c.550delA, p.Ile184Leufs*32	2	2/2 (100.0 %)	1/2 (50.0%)	NA	NA	1.549	Two generations; two affected and one unaffected
CFH c.578C>T, p.Ser193Leu; *CFH* c.908G>A, p.Arg303Gln	9	6/9 (66.7 %)	4/9 (44.4 %)	6/6 (100.0 %)	4/6 (66.7 %)	0.885	Three families: 1) two generations; four affected and six unaffected. 2) one generation; three affected and two unaffected. 3) two generations; three affected and one unaffected
CFH c.607_610dupCCAA, p.Lys204Thrfs*26	3	3/3 (100.0 %)	2/3 (66.7%)	NA	NA	0.895	One generation; three affected and one unaffected
CFH c.764G>A, p.Gly255Glu	3	2/3 (66.7 %)	2/3 (66.7 %)	2/2 (100.0 %)	2/2 (100.0 %)	0.494	Two families: 1) one generation; two affected. 2) two generations; one affected and three unaffected
CFH c.901delG, p.Ala301Glnfs*22	2	2/2 (100.0 %)	2/2 (100.0 %)	NA	NA	1.436	Two generations; two affected
CFH c.1069T>A, p.Cys357Ser	1	ND	ND	ND	ND	1.083	One generation; one affected and one unaffected
CFH c.1126C>T, p.Gln376*	1	ND	ND	ND	ND	1.402	One generation; two affected and one unaffected
CFH c.1198C>A, p.Gln400Lys	4	1/4 (25.0 %)	0/4 (0.0 %)	1/2 (50.0 %)	0/2 (0.0 %)	0.043	Two generations; one affected and six unaffected
CFH c.1215G>T, p.Lys405Asn	2	1/2 (50.0 %)	0/2 (0.0 %)	ND	ND	1.464	Two generations; one affected and four unaffected
CFH c.1222C>T, p.Gln408*	6	6/6 (100.0 %)	5/6 (83.3 %)	NA	NA	-0.302	Two families: 1) two generations; four affected and three unaffected. 2) three generations; three affected and one unaffected

Supplementary Table 5. Family structure and segregation of rare *CFH* and *CFI* variants in AMD families (continued)

**Variant**	**No. of carriers**	**No. of affected carriers (any AMD)/total no. of carriers (%)**	**No. of affected carriers (late AMD)/total no. of carriers (%)**	**No. of affected carriers (any AMD)/total no. of carriers (%) [age 65 threshold]**	**No. of affected carriers (late AMD)/total no. of carriers (%) [age 65 threshold]**	**GRS carriers, median**	**Family structure**
CFH c.1611T>A, p.His537Gln	1	ND	ND	ND	ND	2.410	Two generations; four affected and thirteen unaffected
CFH c.1697-17_1697-8del	3	1/3 (33.3 %)	1/3 (33.3 %)	ND	ND	-1.019	Two generations; one affected and five unaffected
CFH c.1778T>A, p.Leu593*	2	2/2 (100.0 %)	1/2 (50.0 %)	NA	NA	2.054	One generation; two affected
CFH c.1922T>C, p.Val641Ala	1	ND	ND	ND	ND	-1.268	Two generations; one affected and one unaffected[c]
CFH c.2497G>A, p.Glu833Lys	1	ND	ND	ND	ND	N/A	Two generations; five affected and three unaffected[d]
CFH c.2572T>A, p.Trp858Arg	6	5/6 (83.3 %)	2/6 (33.3 %)	NA	NA	0.117	Two generations; five affected and three unaffected[d]
CFH c.2596+8G>T	2	1/2 (50.0 %)	1/2 (50.0 %)	NA	NA	2.930	Two generations; five affected and one unaffected
CFH c.2669G>T, p.Ser890Ile	1	ND	ND	ND	ND	1.914	One generation; three affected and three unaffected
CFH c.2748C>G, p.Tyr916*	2	2/2 (100.0 %)	1/2 (100.0 %)	NA	NA	N/A	Two generations; two affected
CFH c.2850G>T, p.Gln950His	13	4/13 (30.8 %)	1/13 (7.7 %)	4/5 (80.0 %)	1/5 (20.0 %)	0.904	Four families: 1) two generations; five affected and eleven unaffected. 2) three generations; one affected and five unaffected.[a] 3) two generations; two affected. 4) two generations; seven affected and six unaffected.[e]
CFH c.2867C>T, p.Thr956Met	2	2/2 (100.0 %)	1/2 (50.0 %)	NA	NA	2.862	Two families: 1) two generations; five affected and four unaffected.[f] 2) one generation; one affected and one unaffected
CFH c.3133+4C>G	1	ND	ND	ND	ND	1.381	One generation; one affected and one unaffected
CFH c.3234G>T, p.Arg1078Ser	3	2/3 (66.7 %)	2/3 (66.7 %)	NA	NA	1.120	One generation; two affected and one unaffected
CFI c.1709G>C, p.Ser570Thr	1	ND	ND	ND	ND	1.487	Two generations; one affected and two unaffected[b]
CFI c.1657C>T, p.Pro553Ser	4	3/4 (75.0 %)	2/4 (50.0 %)	3/3 (100.0 %)	2/3 (66.7 %)	1.630	Two families: 1) two generations; five affected and three unaffected.[f] 2) two generations; five affected and four unaffected

Supplementary Table 5. Family structure and segregation of rare *CFH* and *CFI* variants in AMD families (continued)

Variant	No. of carriers	No. of affected carriers (any AMD)/total no. of carriers (%)	No. of affected carriers (late AMD)/total no. of carriers (%)	No. of affected carriers (any AMD)/total no. of carriers (%) [age 65 threshold]	No. of affected carriers (late AMD)/total no. of carriers (%) [age 65 threshold]	GRS carriers, median	Family structure
CFI c.1342C>T, p.Arg448Cys	4	4/4 (100.0 %)	3/4 (75.0 %)	NA	NA	0.982	Two families: 1) two generations; four affected and six unaffected. 2) two generations; three affected and one unaffected
CFI c.1322A>G, p.Lys441Arg	1	ND	ND	ND	ND	1.929	One generation; three affected and one unaffected
CFI c.1217G>A, p.Arg406His	1	ND	ND	ND	ND	1.390	Two generations; one affected and one unaffected[c]
CFI 1016G>A, p.Arg339Gln	3	2/3 (66.7 %)	1/3 (33.3 %)	2/2 (100.0 %)	1/2 (50.0 %)	2.169	Two generations; three affected and two unaffected
CFI c.898G>A, p.Ala300Thr	1	ND	ND	ND	ND	1.264	One generations; two affected
CFI c.392T>G, p.Leu131Arg	5	3/5 (60.0 %)	3/5 (60.0 %)	3/3 (100.0 %)	3/3 (100.0 %)	1.145	Two generations; seven affected and six unaffected[e]
CFI c.563G>C, p.Gly188Ala	5	3/5 (60.0 %)	2/5 (40.0 %)	3/3 (100.0 %)	2/3 (66.7 %)	1.778	Two generations; three affected and four unaffected
CFI c.355G>A, p.Gly119Arg	18	14/18 (77.8 %)	11/18 (61.1 %)	14/14 (100.0 %)	11/14 (78.6 %)	2.784	Ten families: 1) two generations; one affected and four unaffected. 2) one generation; two affected and two unaffected. 3) two generations; three affected and one unaffected. 4) two generations; one affected and one unaffected. 5) one generation; one affected and one unaffected. 6) two generations; two affected and three unaffected. 7) two generations; one affected and two unaffected. 8) two generations; two affected and one unaffected. 9) one generation; five affected. 10) two generations; three affected and two unaffected.
CFI c.148C>G, p.Pro50Ala	1	ND	ND	ND	ND	0.294	Two generations; two affected

Rare complement factor H (*CFH*) and complement factor I (*CFI*) variants identified in AMD families. The fraction of individuals carrying a given rare *CFH* or *CFI* variant that manifests AMD was determined. As advanced AMD stages usually lead to severe vision loss (in contrast to the early and intermediate AMD stages, who generally lead to minimal loss of visual acuity), we also provided the fraction of individuals with a given rare *CFH* or CFI variant that manifests advanced AMD. Furthermore, the family cohort consisted of a substantial proportion of young carriers. As they did not reach the age of onset of AMD, and might still develop the disease, the analysis was repeated by excluding the family members without AMD below the age of 65 years. ND = not determined: If only one carrier of specific *CFH* or *CFI* variant was identified, we could not determine segregation. NA = not applicable: For the sub-analysis of the segregation of rare *CFH* and *CFI* variants with the age threshold of 65 years of age (as described above), sometimes all carriers were 65 years or older, and this sub-analysis for that particular rare *CFH* or *CFI* variant was not applicable. In some families multiple rare *CFH* and/or *CFI* variants were identified. The variants *CFH* p.Ser193Leu and *CFH* p.Arg303Gln always segregated together. Three carriers had two different *CFH* variants that did not segregate together (two individuals carried the variants *CFH* p.Ala161Ser and *CFH* p.Gln950His, one individual carried the variants *CFI* p.Leu131Arg and *CFH* p.Gln950His), and therefore were analyzed separately. Consequently, the total number of carriers in the table is 135 instead of 132. Numbers [a-f] indicate the same families. In total there are 51 families carrying rare variants in the *CFH* or *CFI* genes. AMD = age-related macular degeneration; GRS = genetic risk score; CFH = complement factor H; CFI = complement factor I.
